# Applying AI to Structured Real-World Data for Pharmacovigilance Purposes: Scoping Review

**DOI:** 10.2196/57824

**Published:** 2024-12-30

**Authors:** Stella Dimitsaki, Pantelis Natsiavas, Marie-Christine Jaulent

**Affiliations:** 1 Laboratoire d'Informatique Médicale et d'Ingénierie des Connaissances en e-Santé - LIMICS Inserm, Université Sorbonne Paris-Nord Sorbonne Université Paris France; 2 Centre for Research and Development Hellas Institute of Applied Biosciences Thessaloniki Greece

**Keywords:** pharmacovigilance, drug safety, artificial intelligence, machine learning, real-world data, scoping review

## Abstract

**Background:**

Artificial intelligence (AI) applied to real-world data (RWD; eg, electronic health care records) has been identified as a potentially promising technical paradigm for the pharmacovigilance field. There are several instances of AI approaches applied to RWD; however, most studies focus on unstructured RWD (conducting natural language processing on various data sources, eg, clinical notes, social media, and blogs). Hence, it is essential to investigate how AI is currently applied to structured RWD in pharmacovigilance and how new approaches could enrich the existing methodology.

**Objective:**

This scoping review depicts the emerging use of AI on structured RWD for pharmacovigilance purposes to identify relevant trends and potential research gaps.

**Methods:**

The scoping review methodology is based on the PRISMA (Preferred Reporting Items for Systematic Reviews and Meta-Analyses) methodology. We queried the MEDLINE database through the PubMed search engine. Relevant scientific manuscripts published from January 2010 to January 2024 were retrieved. The included studies were “mapped” against a set of evaluation criteria, including applied AI approaches, code availability, description of the data preprocessing pipeline, clinical validation of AI models, and implementation of trustworthy AI criteria following the guidelines of the FUTURE (Fairness, Universality, Traceability, Usability, Robustness, and Explainability)-AI initiative.

**Results:**

The scoping review ultimately yielded 36 studies. There has been a significant increase in relevant studies after 2019. Most of the articles focused on adverse drug reaction detection procedures (23/36, 64%) for specific adverse effects. Furthermore, a substantial number of studies (34/36, 94%) used nonsymbolic AI approaches, emphasizing classification tasks. Random forest was the most popular machine learning approach identified in this review (17/36, 47%). The most common RWD sources used were electronic health care records (28/36, 78%). Typically, these data were not available in a widely acknowledged data model to facilitate interoperability, and they came from proprietary databases, limiting their availability for reproducing results. On the basis of the evaluation criteria classification, 10% (4/36) of the studies published their code in public registries, 16% (6/36) tested their AI models in clinical environments, and 36% (13/36) provided information about the data preprocessing pipeline. In addition, in terms of trustworthy AI, 89% (32/36) of the studies followed at least half of the trustworthy AI initiative guidelines. Finally, selection and confounding biases were the most common biases in the included studies.

**Conclusions:**

AI, along with structured RWD, constitutes a promising line of work for drug safety and pharmacovigilance. However, in terms of AI, some approaches have not been examined extensively in this field (such as explainable AI and causal AI). Moreover, it would be helpful to have a data preprocessing protocol for RWD to support pharmacovigilance processes. Finally, because of personal data sensitivity, evaluation procedures have to be investigated further.

## Introduction

### Background

Pharmacovigilance is defined by the World Health Organization as “the science and activities relating to the detection, assessment, understanding, and prevention of adverse effects or any other drug-related problem” [1]. Pharmacovigilance plays a crucial role in ensuring the safety of medications and protecting the health of patients because it mostly focuses on the identification of potential adverse drug reactions (ADRs) after medicinal products have been licensed and released to the public.

ADRs can range from mild and tolerable side effects to severe and life-threatening events. They constitute 5% to 7% of emergency department consultations [2]. Their impact in terms of public health is significant because there are estimates concluding that ADRs can cause an increase in the duration of hospitalization stays for outpatient (mean 9.2, SD 0.2 d) and inpatient (mean 6.1, SD 2.3 d) settings [3]. Typically, pharmacovigilance professionals analyze data from individual case safety report (ICSR) databases (such as the Food and Drug Administration Adverse Event Reporting System, the database maintained by the US Food and Drug Administration) to identify potential pharmacovigilance signals, namely potential causal relationships between an ADR and a drug. ICSRs are typically submitted either by patients or by health care or pharmacovigilance professionals, and they are the main data source used today for pharmacovigilance. However, ICSR databases are subject to many biases; in addition, underreporting has been identified as a huge issue [4]. Moreover, such databases frequently lack information that could make a significant difference in the examination of a potential signal (eg, patients’ medical history). Hence, the early detection of potential pharmacovigilance signals by collecting and analyzing data from various sources is critical to prevent serious side effects as soon as possible.

The term “real-world data” (RWD) refers to data collected outside of the controlled environment of clinical trials, such as electronic health records (EHRs), patient registries, insurance claims databases, electronic prescription systems, and so on. There is a growing interest in using RWD for pharmacovigilance signal management to facilitate faster and more efficient postmarketing surveillance [5]. The significance of RWD in pharmacovigilance lies in its potential for representing longitudinal real-world patient experiences and health care practices that can provide insights into drug safety under real-life conditions. Analyzing RWD could also enrich and consolidate the already existing knowledge on ADRs (eg, by detecting new cofounders). Indicatively, a federated RWD network was used recently to validate the value of RWD in terms of pharmacovigilance signal management [6].

To this end, the European Medicines Agency and the US Food and Drug Administration have established infrastructures for the leverage of RWD for drug safety purposes, called Data Analysis and Real World Interrogation Network (DARWIN) [7] and the Sentinel Initiative [8], respectively. RWD are also being actively investigated for purposes beyond drug safety (eg, epidemiology) [9]. It should be noted that although RWD could in principle provide a good overview of patients’ clinical course, two major challenges are preventing their use: (1) these datasets typically come with significant data quality risks and usually contain a high proportion of null values and errors; and (2) because of legal, ethical, and regulatory issues (eg, patient privacy issues), it is difficult to access these data sources.

### Rationale

Artificial intelligence (AI) is widely acknowledged as a potentially very useful technical breakthrough that could be used to support decisions in health care (eg, clinical decision support systems) due to its ability to efficiently process big data to seek useful information. AI could be used to identify patterns and associations within large amounts of data (eg, RWD) where traditional statistical methods of data analysis may struggle to extract because of the amount and complexity (eg, nonlinear relationships between variables) of the data. AI has been widely investigated regarding its applications in health care (eg, personalized medicine) with promising results [10,11]; however, it is not yet widely applied in clinical practice. In the context of pharmacovigilance, AI could potentially support multiple aspects (eg, the identification of patient subpopulations who may be more vulnerable to specific ADRs), contributing to the vision of personalized drug safety management.

### Objectives

The objective of this scoping review (SR) was to identify and characterize the current research trends regarding the use of AI on structured RWD for pharmacovigilance and identify relevant gaps.

## Methods

The PRISMA (Preferred Reporting Items for Systematic Reviews and Meta-Analyses) [12] methodology or rationale was applied. The PRISMA-ScR (Preferred Reporting Items for Systematic Reviews and Meta-Analyses extension for Scoping Reviews) statement is a road map for authors to describe more precisely the state of the art and the findings of the literature search, as well as discuss the results.

### Eligibility Criteria

Journal and conference articles written in English were selected if they focused on pharmacovigilance and reported the use of symbolic and nonsymbolic AI approaches applied to RWD, specifically EHRs, insurance claims databases, and administrative health data (Textbox 1).

**Inclusion and exclusion criteria for the scoping review.**

**Inclusion criteria**

- Article type: research
- Language: English
- Data type: tabular
- Data analysis method: symbolic artificial intelligence (AI) and nonsymbolic AI

**Exclusion criteria**

- Article type: review and opinion articles
- Language: other
- Data type: image and text
- Data analysis method: statistical

Review and opinion articles were excluded from the final manuscript selection. Furthermore, research articles focusing on image and text data (eg, social media and clinical notes) were also excluded. In addition, AI methods focusing on the use of natural language processing (NLP), natural language understanding, image processing, or object detection were considered beyond the scope of this work.

A key issue that came up during this SR was the lack of a clear distinction between plain statistical methods and machine learning (ML) approaches because these 2 domains frequently overlap, and these 2 terms are sometimes used interchangeably. In this manuscript, we acknowledge that the difference between AI and statistical methods is that AI creates models that can “learn” from data during iterative training processes, while statistical methods deal with finding relationships between variables. Thus, we considered the iterative “learning” part of an algorithm as the key feature to classify the algorithm as AI and ML. We excluded papers that were based on algorithms with no iterative “learning” scheme because we considered them to be part of the “plain statistical methods” approaches. Finally, we excluded papers that focused on adverse drug events related to medical devices.

### Information Sources and Search Strategy

A search query was developed and executed on January 31, 2024, to include research articles from 2010 to 2024 exclusively from the MEDLINE scientific library, given that it is the oldest and biggest repository of journal articles in life sciences. Textbox 2 presents the query structure.

**Query structure ([pharmacovigilance terms with OR] AND [artificial intelligence (AI) terms with OR] AND [real-world data (RWD) terms with OR]).**

**Pharmacovigilance (keywords relevant to known adverse drug reaction [ADR] categories, synonyms of drug safety, pharmacovigilance terminology, and known individual case safety report [ICSR] databases)**

V OR “pharmacovigil*” OR “pharmaco-vigil*” OR “side effect*” OR “adverse reaction*” OR “Product Surveillance” OR “postmarket*” OR pharmacoepidemiol* OR pharmaco-epidemiol* OR “drug safety” OR “drug event*” OR “toxicit*” OR “drug reaction*” OR “adverse drug*” OR “allerg*” OR “post-market*” OR “post market*” OR vaccinovigil* OR vaccino-vigil* OR eudravigilance OR “individual case safety report*” OR ICSR OR VAERS OR FAERS OR AERS OR vigibase OR “adverse effect*” OR “adverse event*” OR hypersensitiv* OR “spontaneous report*” OR “yellow card” OR “yellow-card” OR ADR OR “personalized pharmacovigilance” OR “precision pharmacovigilance” OR “pharmacosurveillance” OR “pharmaco-surveillance”

**AI (categories of AI, terms that are used in the development of an AI model, explainable and interpretable AI methods, and different AI architectures)**

“artificial intelligence” OR AI OR “machine learning” OR ML OR “neural network*” OR NN* OR “deep learning” OR DL OR ontolog* OR “knowledge engineering” OR KE OR reasoning OR inference OR “semantic web” OR “OWL” OR “Web Ontology Language” OR SWRL OR “RDF” OR “Resource Description Framework” OR “prediction” OR “estimation” OR “XAI” OR “SHAP” OR “Shapley value” OR “LIME” OR “Local Interpretable Model-agnostic Explanations” OR “DeepSHAP” OR “DeepLIFT” OR “CXplain” OR “Explainable Artificial Intelligence” OR “Explainable machine learning” OR “Interpretable artificial intelligence” OR “Interpretable machine learning”

**RWD or real-world evidence (categories of RWD and data models that are used to store RWD)**

“Real World Evidence” OR “Real World Data” OR RWE OR RWD OR “Observational Medical Outcomes Partnership” OR “OMOP” OR “Electronic Healthcare Record*” OR “EHR” OR “Electronic Medical Record*” OR “EMR*” OR EHDEN OR OHDSI OR i2b2 OR Sentinel OR DARWIN OR “Data Analysis and Real World Interrogation Network” OR administrative OR claim* OR “Observational Health Data Sciences and Informatics” OR “European Health Data Evidence Network” OR “multimodal data” OR “multimodal drug data” OR “multidimensional data” OR “multidimensional drug data” OR “multi-modal data” OR “multi-modal drug data” OR “multi-dimensional data” OR “multi-dimensional drug data”

### Selection Process

The initial phase (phase 1) focused on screening the titles and abstracts of the articles retrieved from the search query (Textbox 2) to map those that potentially met our inclusion criteria and exclude irrelevant studies using the Rayyan tool (Rayyan Systems Inc) [13]. Rayyan is an AI tool designed to facilitate remote collaboration among researchers when conducting systematic literature reviews. The platform gathers the titles and abstracts of all articles selected for the study, and reviewers can evaluate the eligibility (ie, “include,” “exclude,” or “maybe”) of every article based on their review’s objectives in blind mode, that is, each reviewer assesses the articles without prior knowledge of the other reviewers’ decisions. We resolved any conflicts that arose during this process through consensus meetings involving all reviewers.

The second phase focused on the full-text review of the papers selected during phase 1 to decide on the final set for inclusion in this study. In the full-text review of the studies selected based on titles and abstracts, we excluded research papers that did not meet ≥1 of the inclusion criteria (ie, strong focus on AI, RWD, and pharmacovigilance) as well as studies that met the exclusion criteria (eg, studies related to image and text data or those following only statistical approaches).

### Data-Charting Process

A standard data extraction form was used to obtain an overview of the 36 selected studies (Tables S1 and S2 in Multimedia Appendix 1). For each study, we extracted information about the authors; journal name (where the study was published); publication year; country of origin (where the study was conducted); the objective of the study; types of organizations that participated in the study (based on the authors’ affiliations); and key findings that relate to the scoping review question, which are described in the next subsection (Data Collection Process and Mapping). Any inconsistencies were discussed and resolved among the reviewers.

### Data Collection Process and Mapping

The selected studies were further elaborated and mapped against evaluation criteria using a spreadsheet. The main categories of mapping criteria were as follows: pharmacovigilance objectives (drug safety core activities and drug safety special topics), data provenance (data source categories and data sources), countries of origin, AI algorithm categories, data preprocessing methods, the use of explainable AI (XAI) methods, code availability, the use of models in clinical practice, ethical AI, and so on. Table 1 presents an external description of the mapping criteria.

**Table 1:** The categories and subcategories used in the risk-of-bias assessment of included studies designed to characterize artificial intelligence (AI) studies on structured real-world data in pharmacovigilance.

Category of bias	Explanation	Subcategories
Selection bias	The bias that occurs when the input data of an AI model underrepresent the target population	Underrepresentation of certain demographic groups Overrepresentation of adverse drug events from specific health care systems or regions
Measurement bias	How the different features are collected and measured	Inconsistent adverse drug event reporting practices Variations in diagnostic criteria or coding practices for medical conditions
Temporal bias	How the study processes time-dependent features	Changes in prescribing patterns or drug formulations over time Seasonal variations in disease prevalence or reporting behaviors
Algorithmic bias	The biases produced form AI model outputs	Differential performance in adverse drug event detection across patient subgroups Biased risk assessments for certain medications or populations
Implicit bias	How stereotypes influence the AI model design and interpretation	Overlooking potential drug interactions more common in specific ethnic groups Underestimating the severity of side effects reported by certain demographics
Confounding bias	How unaccounted-for confounders influence prediction	Failing to consider comorbidities when assessing drug safety profiles Not accounting for polypharmacy effects in adverse-event analysis
Automation bias	This refers to the tendency to overly rely on automated systems	Overlooking rare or unusual adverse drug events not flagged by AI systems Reduced critical evaluation of AI-generated safety signals by human experts

### Studying Risk-of-Bias Assessment

To effectively map the risk of bias in each included study, we considered selection, measurement, temporal, implicit, confounding and automation biases. Furthermore, we translated these categories into more specific categories according to our study (Table 1).

### Synthesis Methods

The mapping strategy was designed based on the 3 main pillars of the objective; in addition, we included general information about the research papers (Table 2). Furthermore, we included free-text fields in the mapping Microsoft Excel file to add significant extra details that cannot be easily classified. These fields included “objective,” “methods,” “assessment,” and “interesting results.” The criteria encompassed specific attributes (eg, drug safety core activities) that were defined based on previous experience of conducting an SR in the field [14] and key interest aspects identified during the review.

Furthermore, in terms of ethical AI, the included studies were evaluated based on trustworthy AI guidelines for solutions in medicine and health care from the FUTURE (Fairness, Universality, Traceability, Usability, Robustness, and Explainability)-AI initiative [15]. These guidelines are separated into 7 categories (fairness, universality, traceability, usability, robustness, explainability, and a general category). For our evaluation procedure, we included only the highly recommended subcategories from each of the 7 main categories for proof of concept (low technology readiness levels for ML models) [16]. Table 3 presents the selected criteria and their description.

**Table 2:** Mapping criteria architecture for each different category in the query. There are 2 types of criteria: textual and binary (yes or no).

Categories and critera		Subcriteria
**General information**		
	PubMed and MEDLINE ID (number)	ID number of articles
	Authors (text)	List of authors
	Title (text)	Article title
	Journal (text)	ID number of articles
	Year published (number)	Year of article publication
	Types of organizations (text)	Types of organizations based on the authors’ affiliation; possible values: health care, government, academia, industry, pharmacovigilance monitoring
	Country (text)	Country where the research was conducted based on the authors’ affiliations
**Pharmacovigilance**		
	Drug safety core activities (text)	Possible values: ADE^a^ detection, ADE monitoring, ADE prevention, ADE assessment, ADE information collection, and ADE reporting
	Drug safety special topics (text)	Possible values: comparative drug analysis, drug interactions, MoA^b^ identification and analysis, personalized drug safety, signal detection, specific (class of) disease, specific (class of) drugs, specific adverse effect, and vaccine safety
	Drug (text)	Drugs being examined in the research papers
	Reaction (text)	Reactions being examined in the research papers
	Indication (text)	Indications being examined in the research papers
	Reference terminologies (text)	Known health informatics terminologies that are detected in the research papers
**AI^c^**		
	AI categories (text)	Possible values: nonsymbolic AI and symbolic AI
	Nonsymbolic AI (text)	Possible values: classification and regression
	Classification (text)	Possible values: random forest, logistic regression, artificial neural network, XGBoost^d^, support vector machine, decision tree, knowledge graph, k-nearest neighbors, gradient boost, naïve Bayes, random survival forest, and extra tree
	Regression (text)	Possible values: logistic regression, linear regression, LASSO^e^, and regularized Cox regression
	Data preprocessing type (text)	Possible values: dimensionality reduction, feature engineering, null imputation, and data cleansing
	Data cleansing (text)	Possible values: data normalization and remove null values
	Feature engineering (text)	Possible values: one-hot encoding, binning, splitting, and calculated features
	Null imputation (text)	Possible values: regression or classification imputation
	Explainable AI methods (text)	Possible values: LIME^f^ and SHAP^g^
	Knowledge representation formalism (text)	Possible values: OWL^h^ and RDF^i^
	Knowledge engineering core activities (text)	Possible values: knowledge extraction, knowledge integration, and knowledge representation
**Real-world data**		
	Data source categories (text)	Possible values: ADE databases, clinical narratives, clinical trials drug information databases, drug regulation documentation, EHRs^j^, genetics and biochemical databases, spontaneous reporting systems, dispensing records from pharmacies, and administrative claims data
	Data source or sources (text)	Possible values: proprietary closed data sources (eg, specific hospital EHR), FAERS^k^, SIDER^l^, SMILES^m^, UK Biobank, Osteoarthritis Initiative dataset, PharmGKB^n^, TwoSIDES, EU-ADR^o^ reference set, Stockholm Electronic Patient Record Corpus, MIMIC^p^, OMIM^q^, DisGeNet^r^, and AEOLUS^s^
	Data model (text)	Possible values: OMOP-CDM^t^, Sentinel, and custom
**Evaluation criteria**		
	Code availability (text)	The availability of the code in an open registry; possible values: yes and no
	Data preprocessing	Information about the data preprocessing procedures; possible values: yes and no
	Clinical use	Information about the evaluation of the produced work pipeline in clinical environments; possible values: yes and no

^a^ADE: adverse drug event.

^b^MoA: mechanism of action.

^c^AI: artificial intelligence.

^d^XGBoost: extreme gradient boosting.

^e^LASSO: least absolute shrinkage and selection operator.

^f^LIME: local interpretable model-agnostic explanations.

^g^SHAP: Shapley additive explanations.

^h^OWL: Web Ontology Language.

^i^RDF: resource description framework.

^j^EHR: electronic health record.

^k^FAERS: Food and Drug Administration Adverse Event Reporting System.

^l^SIDER: Side Effect Resource.

^m^SMILES: Simplified Molecular Input Line Entry System.

^n^PharmGKB: Pharmacogenomics Knowledge Base.

^o^EU-ADR: European Union Adverse Drug Reaction.

^p^MIMIC: Medical Information Mart for Intensive Care.

^q^OMIM: Online Mendelian Inheritance in Man.

^r^DisGeNet: gene-disease association network.

^s^AEOLUS: Adverse Event Open Learning through Universal Standardization.

^t^OMOP-CDM: Observational Medical Outcomes Partnership Common Data Model.

**Table 3:** Detailed description of the FUTURE (Fairness, Universality, Traceability, Usability, Robustness, and Explainability)-AI highly recommended and proof-of-concept machine learning guidelines used for this study, along with a general category (Table S3 Multimedia Appendix 1).

Categories and recommendations		Description
**Fairness**		
	Define sources of bias	Identification of possible types and sources of bias for the AI^a^ tool during the design phase (eg, sex, gender, age, ethnicity, socioeconomics, geography, comorbidities or disability of patients, and human biases during data labeling)
**Universality**		
	Define clinical settings	Specification of the clinical settings in which the AI tool will be applied (eg, primary health care centers, hospitals, home care, low- vs high-resource settings, and 1 country or multiple countries)
	Evaluate using external data	Testing of the developed AI model to an external dataset with different characteristics from the training set
**Traceability**		
	Provide documentation (eg, technical and clinical)	Creation of documentation files that provide technical (eg, public repositories) and clinical information (eg, bias of the model based on its use)
**Usability**		
	Define user requirements	Specification of the model’s use from health care professionals
**Robustness**		
	Define sources of data variation	Specification of data sources’ variation that may impact the AI tool’s robustness in the real world (differences in equipment, technical fault of the machine, data heterogeneities during data acquisition or annotation, or adversarial attacks)
	Train with representative data	Data for the training process should represent the population based on the case study for which the AI model has been developed
	Evaluate and optimize robustness	Risk mitigation measures should be implemented to optimize the robustness of the AI model, such as regularization, data augmentation, data harmonization, or domain adaptation
**Explainability**		
	Define explainability needs	Use of interpretable or explainable models
**General**		
	Engage interdisciplinary stakeholders throughout the AI lifecycle	—^b^
	Implement measures for data privacy and security	—
	Define adequate evaluation plan (eg, datasets, metrics, and reference methods)	—

^a^AI: artificial intelligence.

^b^Not applicable.

### Reporting Risk-of-Bias Assessment

The selection of only studies written in English and the exclusion of AI studies focused on text mining or NLP, image processing, and statistical analysis could be identified as potential risks for bias. Furthermore, the selection of papers only from the MEDLINE database could be identified as a potential bias risk because it potentially leads to the omission of papers from other databases (eg, AI databases).

## Results

### Study Selection

**Figure 1:**
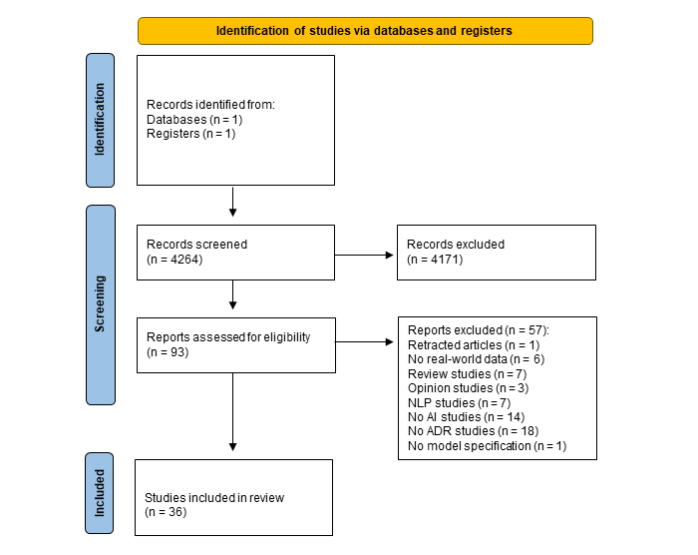
PRISMA (Preferred Reporting Items for Systematic Reviews and Meta-Analyses) flowchart. ADR: adverse drug reaction; AI: artificial intelligence; NLP: natural language processing.

The PubMed search query originally returned 4264 studies. During the abstract and title screening process (phase 1), we selected 93 (2.18%) of the 4264 articles for full-text screening (phase 2). During phase 2, based on the inclusion criteria, of these 93 research papers, 36 (39%) were selected. The PRISMA-ScR flowchart (Figure 1) presents a detailed overview of the selection procedure. The PRISMA-ScR checklist is presented in Multimedia Appendix 1.

### Study Characteristics

The included studies were published between 2015 and 2023, with a notable increase in the number of studies after 2019 (Table 4).

Of the 36 studies, 19 (53%) originated from the United States, 4 (11%) from Korea, and 4 (11%) from the United Kingdom, while the rest of the studies (n=9, 25%) were distributed across a variety of other countries (Table 5).

Most of the studies (30/36, 83%) were conducted from academia (Table 6).

**Table 4:** The distribution of studies through the years (n=36).

Years	Studies, n (%)
2015	1 (3)
2016	2 (5)
2017	3 (8)
2018	2 (5)
2019	2 (5)
2020	4 (11)
2021	10 (29)
2022	7 (20)
2023	5 (14)

^a^Includes studies conducted in multiple countries.

**Table 5:** Country of origin of the included studies (n=36)^a^.

Countries	Studies, n (%)
United States	19 (53)
South Korea	4 (11)
United Kingdom	4 (11)
Canada	3 (8)
Sweden	3 (8)
China	3 (8)
France	3 (8)
Australia	2 (6)
Netherlands	2 (6)
Bangladesh	1 (3)
Israel	1 (3)
Belgium	1 (3)
Denmark	1 (3)
Taiwan	1 (3)
Ireland	1 (3)
Switzerland	1 (3)

^a^Includes studies that involved >1 type of organization.

**Table 6:** Types of organizations that participated in the including studies (n=36)^a^.

Organizations	Studies, n (%)
Academia	30 (83)
Health care	9 (25)
Industry	6 (17)
Government	2 (6)
Regulatory bodies	1 (3)

^a^Includes studies that involved multiple databases.

^b^EHR: electronic health record.

^c^SRS: spontaneous reporting system.

^d^ADE: adverse drug event.

In terms of AI, of the 36 studies, 34 (94%) applied only nonsymbolic AI, and 1 (3%) used only symbolic AI, while 1 (3%) study combined the symbolic and nonsymbolic AI technical paradigms. Of the 34 nonsymbolic AI articles, 29 (85%) used classification tasks, whereas 3 (9%) selected regression algorithms, 3 (9%) applied causality algorithms (causal inference: n=2, 67%; causal discovery: n=1, 33%), and only 1 (3%) applied an association rule mining technique. The association rule mining study [17] followed a mathematical framework called formal concept analysis to create association rules between drugs and phenotypes to detect possible ADRs. Moreover, of the 29 studies that used classification tasks, 6 (21%) used XAI techniques, of which 4 (67%) used Shapley additive explanations, 1 (17%) used local interpretable model-agnostic explanations, and 1 (17%) tested both approaches.

Regarding RWD (Table 7), of the 36 articles, 28 (78%) focused on the use of EHRs (from local hospital databases), 4 (11%) used data from pharmacy dispensing records, and 3 (8%) used administrative claims data, while 2 (6%) focused on patient registries and 1 (3%) on insurance claims. In addition, a variety of other sources were used, including RWD such as drug information databases (3/36, 8%), spontaneous reports (3/36, 8%), adverse drug event databases (2/36, 6%), electronic prescription data (2/36, 6%), and genetics and biochemical databases (1/36, 3%).

Of the 36 studies, 23 (64%) used AI for ADR detection, 4 (11%) examined ADR assessment, 2 (6%) focused on ADR monitoring, 7 (19%) investigated ADR prevention, and 2 (6%) used AI to collect information about ADRs (Table 8).

**Table 7:** Variety of data used in the development of artificial intelligence models in the included studies (n=36)^a^.

Type of database	Studies, n (%)
EHRs^b^	28 (78)
Drug information databases	4 (11)
Dispensing records from pharmacies	4 (11)
SRSs^c^	3 (8)
Administrative claims data	3 (8)
Patient registries	2 (6)
Electronic prescription data	2 (6)
ADE^d^ databases	2 (6)
Insurance claims	1 (3)

^a^Includes studies that examined multiple pharmacovigilance core activities.

^b^ADR: adverse drug reaction.

**Table 8:** Description of pharmacovigilance core activities in the included studies (n=36)^a^.

Pharmacovigilance core activities	Studies, n (%)
ADR^b^ detection	23 (64)
ADR prevention	7 (19)
ADR assessment	4 (11)
ADR monitoring	2 (6)
ADR information collection	2 (6)

^a^Includes studies that involved multiple AI algorithms.

^b^XGBoost: extreme gradient boosting.

^c^LASSO: least absolute shrinkage and selection operator.

^d^No algorithms.

The classification studies (29/36, 81%; Table 9) tested several AI techniques, with random forest (RF) being the most frequently used algorithm (17/29, 59%). However, the regression studies (3/36, 8%) developed AI models only with extreme gradient boosting (1/3, 33%) and logistic regression (2/3, 67%).

Finally, for the evaluation of AI models (Table 10), most of the studies (24/36, 67%) reported area under the receiver operating characteristic curve as the primary metric.

Of the 36 studies, 32 (89%) investigated specific drug safety topics: 16 (50%) on specific adverse effects, 14 (44%) on specific class of drugs, 8 (25%) on specific (class of) diseases, 6 (19%) on signal detection, 3 (9%) on drug interactions, 2 (6%) on personalized drug safety, and 1 (3%) on vaccine safety (Table 11).

**Table 9:** The types of artificial intelligence (AI) algorithms that the models developed in the included studies (n=36).

AI models and algorithms		Studies, n (%)
**Classification (n=29)** ^a^		
	Random forest	17 (59)
	XGBoost^b^	10 (34)
	Artificial neural network	8 (28)
	Logistic regression	8 (28)
	Support vector machine	7 (24)
	Decision tree	5 (17)
	K-nearest neighbor	2 (7)
	Gradient boost	2 (7)
	LASSO^c^	2 (7)
	Extra tree	1 (3)
	Naïve Bayes	1 (3)
	Random survival forest	1 (3)
	Linear regression	1 (3)
	Regularized Cox regression	1 (3)
**Regression (n=3)**		
	XGBoost	1 (33)
	Logistic regression	2 (67)
Causality^d^ (n=3)		3 (100)

^a^Includes studies that involved multiple model evaluation metrics.

**Table 10:** Evaluation metrics of artificial intelligence (AI) models developed in the included studies (n=36)^a^.

AI model evaluation metrics	Studies, n (%)
Area under the receiver operating characteristic curve	24 (67)
Accuracy	10 (28)
*F*_1_-score	8 (22)
Precision	11 (31)
Recall	10 (28)
Negative predictive value	6 (17)
Sensitivity	9 (25)
Specificity	7 (19)
Other (≤2)	20 (56)

^a^Includes studies that examined multiple pharmacovigilance topics.

**Table 11:** Specialized pharmacovigilance topics presented in the included studies (n=36)^a^.

Specialization of pharmacovigilance topics	Studies, n (%)
Specific adverse effect	16 (50)
Specific (class of) drugs	14 (44)
Specific (class of) disease	8 (25)
Signal detection	6 (19)
Drug interactions	3 (9)
Personalized drug safety	2 (6)
Vaccine safety	1 (3)

^a^Includes studies that used multiple data sources.

^b^SIDER: Side Effect Resource.

^c^FAERS: Food and Drug Administration Adverse Event Reporting System.

Table 12 presents the diversity in the data sources used in the included studies. Of the 36 studies, 29 (81%) chose proprietary closed data sources (eg, specific hospital EHRs) for their experiments. Along with EHR data, other data sources were also used (eg, Food and Drug Administration Adverse Event Reporting System and Side Effect Resource). Of the 36 studies, 2 (6%) selected the Stockholm Electronic Patient Record Corpus. The remaining RWD sources (Medical Information Mart for Intensive Care and the Osteoarthritis Initiative dataset) are represented in only 2 (6%) of the 36 studies (n=1, 50% for every database).

In terms of data models, of the 36 studies, 27 (75%) used proprietary data models, 3 (8%) did not mention any data model, 5 (14%) used the Observational Medical Outcomes Partnership Common Data Model (OMOP-CDM), and 1 (3%) used the Sentinel model (Table 13).

**Figure 2:**
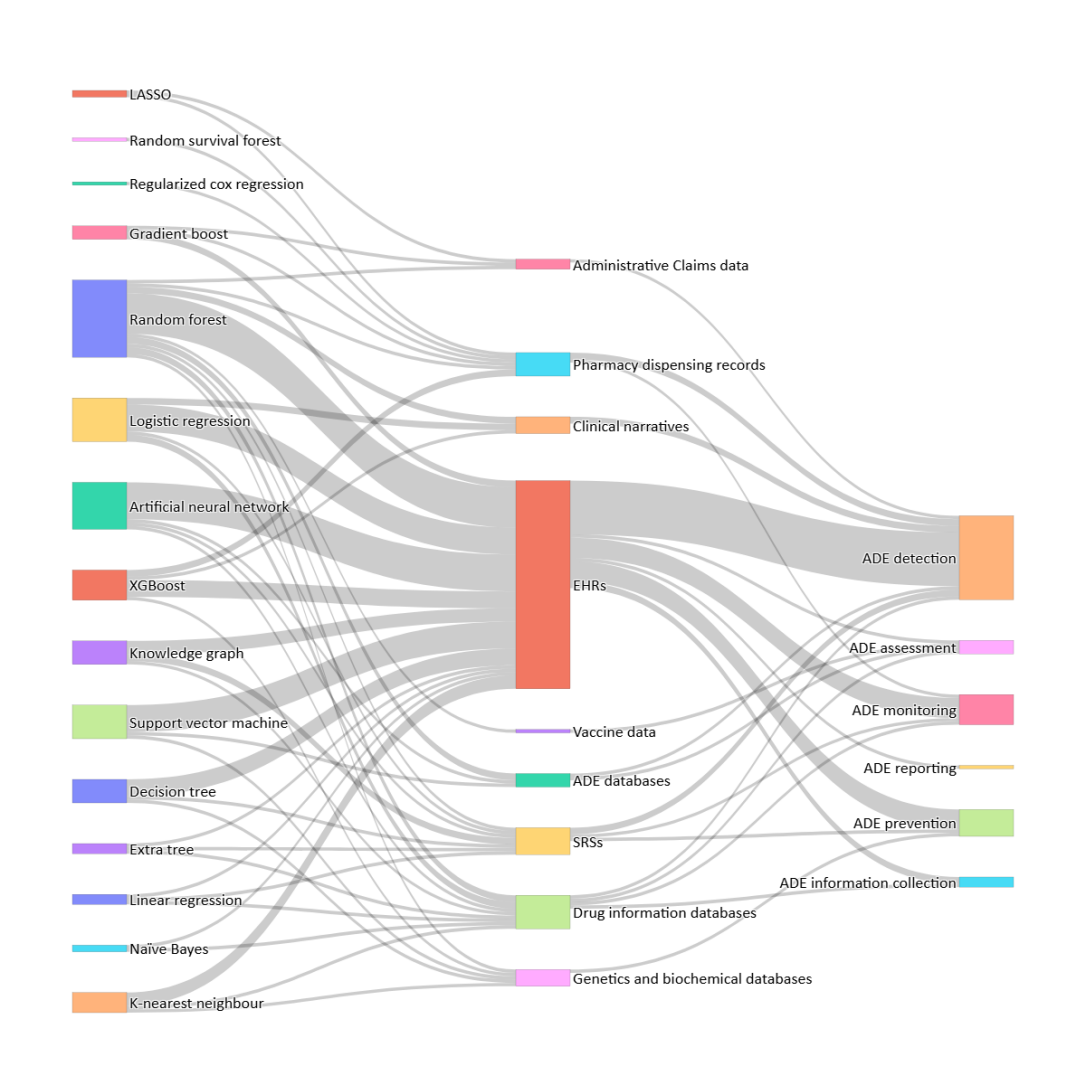
Association pathways between artificial intelligence models, data sources, and drug safety categories in the included studies. ADE: adverse drug event; EHR: electronic health record; LASSO: least absolute shrinkage and selection operator; SRS: spontaneous reporting system; XGBoost: extreme gradient boosting.

**Figure 3:**
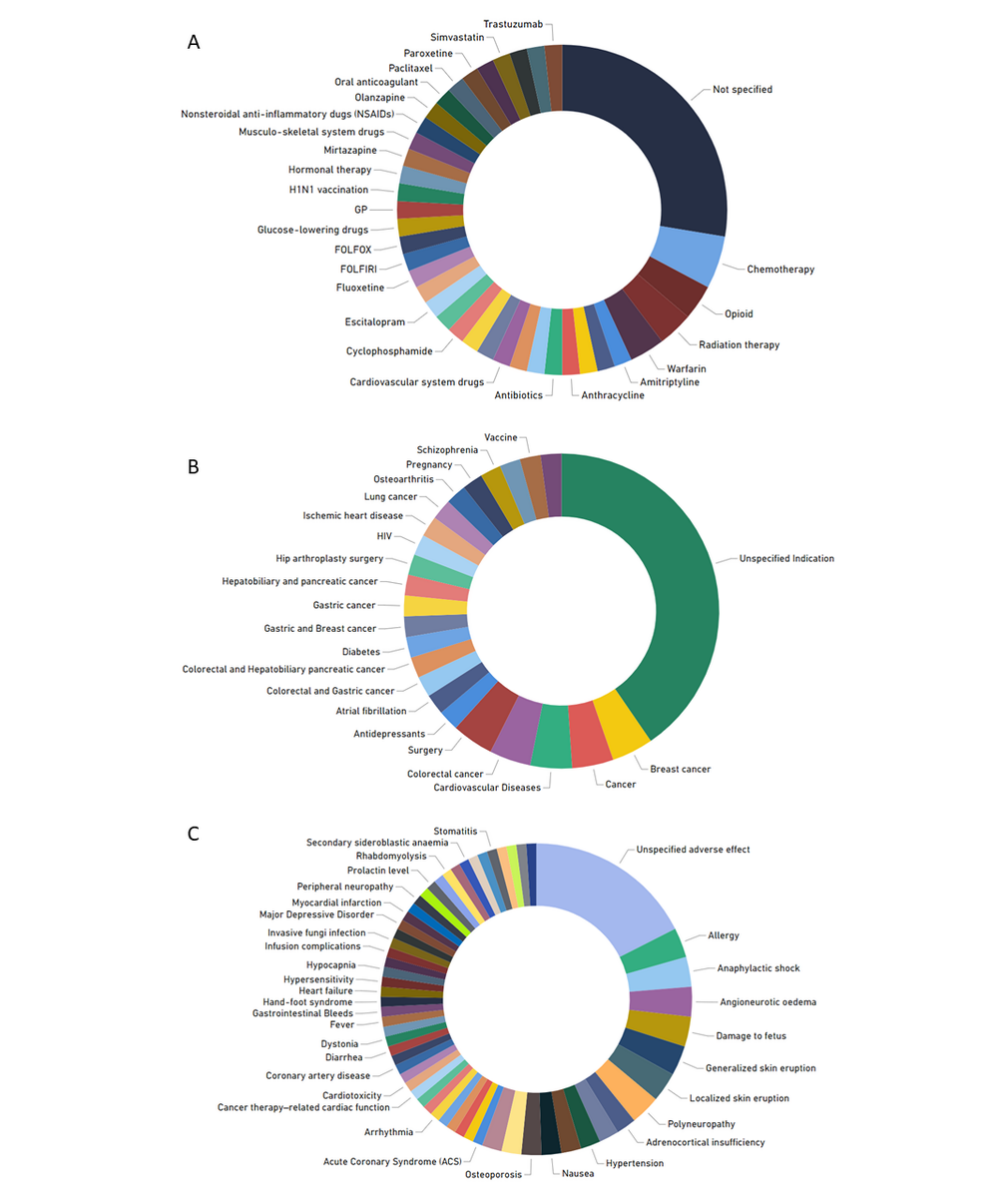
Drugs, indications, and reactions in the included studies. (A) Drugs. (B) Indications. (C) Reactions.

Figure 2 presents the case studies examined in the included articles. Notably, an important number of studies (20/36, 55%) did not work in specific ADR case studies. Another significant outcome is the diversity of case studies; the articles do not focus on a specific drug, indication, or reaction. It can be observed that chemotherapy drugs and their associated reactions in various types of cancers emerge as slightly more prominent categories in this review (Figure 3).

**Table 12:** Variety of data sources used in the included studies (n=36)^a^.

Data sources	Studies, n (%)
Proprietary closed data sources	29 (81)
Other	12 (33)
SIDER^b^	3 (8)
FAERS^c^	2 (6)
Stockholm Electronic Patient Record Corpus	2 (6)

^a^OMOP-CDM: Observational Medical Outcomes Partnership Common Data Model.

**Table 13:** Included studies’ distribution based on data models that the data are stored (n=36).

Data models	Studies, n (%)
Custom	27 (75)
OMOP-CDM^a^	5 (14)
Unknown	3 (8)
Sentinel	1 (3)

Although most of the studies (21/36, 58%) used complex AI algorithms (black boxes), such as RF (an ensemble method) and artificial neural networks (ANNs), to construct their prediction models in all ADR categories, many studies (15/36, 42%) used simple interpretable ML approaches such as logistic regression. Moreover, it is important to highlight that all studies worked on EHR databases, except for the adverse drug event assessment category in which we detected a single study with a vaccine database.

RWD databases were also used alongside other types of data; for example, EHRs were mostly combined with spontaneous reporting systems and drug information databases, vaccine data with adverse drug event databases, and administrative claims data with spontaneous reporting systems. Furthermore, some of the studies (3/36, 8%) integrated different types of observational data to develop AI models, combining pharmacy dispensing records with EHRs and administrative claims data.

### Evaluation Results

Only 3 (8%) of the 36 studies included in this SR openly provided their code. In addition, only 16 (44%) of the 36 studies included a detailed description of data preprocessing pipelines for RWD. Moreover, just 4 (11%) of the 36 studies evaluated their methodology within a clinical environment (Table 14).

**Table 14:** Summary of code availability, data preprocessing, and clinical validation evaluation criteria (n=36).

Evaluation metrics	Studies, n (%)	
	Yes	No
Code availability	3 (8)	33 (92)
Data preprocessing	16 (44)	20 (56)
Clinical validation	5 (14)	31 (86)

In terms of trustworthy AI, only 5 (14%) of the 36 studies scored <50% on the Fairness, Universality, Traceability, Usability, Robustness, and Explainability–AI (FUTURE-AI) criteria (Tables 15 and 16). Among the studies that achieved scores of >75% [18-20], 3 (75%) out of 4 used external data to evaluate their models, addressing the Universality criterion (Table S4 in Multimedia Appendix 1).

**Table 15:** Distribution of included studies according to the FUTURE (Fairness, Universality, Traceability, Usability, Robustness, and Explainability)-AI guidelines (n=36)^a^.

Data models	Studies, n (%)
Custom	27 (75)
OMOP-CDM^a^	5 (14)
Unknown	3 (8)
Sentinel	1 (3)

^a^Includes studies that fell into multiple FUTURE-AI and general categories.

**Table 16:** Evaluation of articles included in the scoping review based on the Fairness, Universality, Traceability, Usability, Robustness, and Explainability–AI (FUTURE-AI) and Code Availability, Data preprocessing and clinical validation criteria.

Study	Year	Code availability	Data preprocessing	Clinical use	FUTURE-AI criteria satisfaction (%)
Anastopoulos et al [18]	2021	No	Yes	No	75
Ward et al [21]	2021	No	Yes	No	67
Zhang et al [22]	2020	No	Yes	No	50
Kim et al [23]	2021	No	No	No	67
Morel et al [24]	2020	Yes	No	Yes	67
Zou et al [25]	2021	No	No	Yes	67
Liu et al [26]	2018	No	No	Yes	42
Zhu et al [27]	2022	No	Yes	No	67
Kidwai-Khan et al [28]	2022	No	Yes	No	58
Sharma et al [29]	2022	No	No	No	58
On et al [30]	2022	No	Yes	No	50
Datta et al [31]	2021	No	No	No	58
Bagattini et al [19]	2019	Yes	Yes	No	75
Gibson et al [32]	2021	No	Yes	No	58
Jeong et al [33]	2018	No	Yes	No	67
Zhao et al [34]	2015	No	Yes	No	58
Zhao and Henriksson [35]	2016	No	Yes	No	42
Segal et al [20]	2019	No	No	Yes	75
Boland et al [36]	2017	No	No	No	67
Wang et al [37]	2021	No	No	No	58
Li et al [38]	2022	No	No	No	58
Jin et al [39]	2020	No	No	No	67
Hansen et al [40]	2016	No	No	No	50
Mosa et al [41]	2021	No	No	No	67
Herrin et al [42]	2021	No	No	No	50
Pichardo et al [43]	2022	No	No	No	67
Puzhko et al [44]	2021	No	No	No	58
Souissi et al [45]	2017	No	No	Yes	42
Personeni et al [17]	2017	No	No	No	42
Zhou et al [46]	2020	Yes	No	No	67
Goyal et al [47]	2023	No	No	No	58
Wang et al [48]	2023	No	Yes	No	58
Hughes et al [49]	2023	No	Yes	No	58
Sharma et al [50]	2023	No	Yes	No	67
Akimoto et al [51]	2023	No	Yes	No	58
Zhang et al [52]	2022	No	Yes	No	58

### Risk of Bias in the Included Studies

Table 17 provides an overview of the distribution of biases across the included studies. Notably, of the 36 studies, 21 (58%) included selection biases, and 17 (47%) included confounding biases. Algorithmic biases were identified in 14 (39%) of the 36 studies. Table S5 in Multimedia Appendix 1 presents the detailed categorization of the included studies in the different risk-of-bias categories.

**Table 17:** Results of the risk-of-bias assessment for each included study organized into subsections that better characterize the artificial intelligence (AI) models developed using structural real-world data in pharmacovigilance.

Bias categories and subcategories			References
**Data-related biases**			
	**Selection bias**		
		Underrepresentation of certain demographic groups	[18,19,21,22,26,28-30,33,38,41,42,44-47,49-52]
		Overrepresentation of adverse drug events from specific health care systems or regions	[18,19,21,22,24,26,28-30,33,38,42,45-47,49-52]
	**Measurement bias**		
		Inconsistent adverse drug event reporting practices	[18,20,21,24,32,38,41,44,45]
		Variations in diagnostic criteria or coding practices for medical conditions	[20,24,32,38,46,51,52]
	**Temporal bias**		
		Changes in prescribing patterns or drug formulations over time	[17,18,21,24,34,39,40,44,45,51,52]
		Seasonal variations in disease prevalence or reporting behaviors	[34,39,40,44,45,51]
**Algorithm-related biases**			
	**Algorithmic bias**		
		Differential performance in adverse drug event detection across patient subgroups	[17,18,27,32,36,37-39,41,45,46,48-50]
		Biased risk assessments for certain medications or populations	[17,18,27,32,36-39,41,45,46,48-50]
	**Implicit bias**		
		Overlooking potential drug interactions more common in specific ethnic groups	[17,18,24,31,41,44]
		Underestimating the severity of side effects reported by certain demographics	[17,24,31,38,41,44]
**Deployment and interpretation biases**			
	**Confounding bias**		
		Failing to consider comorbidities when assessing drug safety profiles	[18,21,22,25,27,31,34,36,44,46-49,51]
		Not accounting for polypharmacy effects in adverse drug event analysis	[18,21,22,25,27,28,31,34,36,40,46-49,51]
	**Automation bias**		
		Overlooking rare or unusual adverse drug events not flagged by AI systems	[19,25,49,50]
		Reduced critical evaluation of AI-generated safety signals by human experts	[19,24,49,50]

### Interesting Results of Individual Studies

It is important to mention that 6% (2/36) of the studies successfully combined AI and a self-controlled case series (SCCS) model for ADR detection. Morel et al [24] introduced the convolutional SCCS (ConvSCCS) model in which the SCCS model is enriched with a convolutional neural network. This allows the ConvSCCS model to consider a few longitudinal data dimensions (eg, drug exposure) from observational data and predict a potential ADR without a prior definition of risk windows, which is mandatory in SCCS models. The ConvSCCS model was tested in glucose-lowering drugs and the risk of bladder cancer case study. Another interesting advantage shown by the results is that the ConvSCCS model is useful for analyzing high-dimensional data while requiring minimal data preprocessing. Zhang et al [22] developed the neural SCCS (NSCCS) model to detect probable drug interactions and control for time-invariant confounders. The NSCCS model was tested in the OMOP-CDM reference dataset [53]. Both the ConvSCCS and NSCCS models outperformed traditional SCCS statistical models in comparative analyses; the ConvSCCS model demonstrated superior precision and computational speed, while the NSCCS model achieved an area under the receiver operating characteristic curve score of 0.779 [22].

Furthermore, Kidwai-Khan et al [28] focused on improving the prediction of preventable adverse events by integrating in an AI decision support tool, EHRs with genetic data (the presence or absence of genes contraindicated with a person’s medication). This is the only study in this review that combined EHRs with genetic data and 1 (25%) of the 4 studies that used XAI methods. In addition, all AI models achieved high evaluation scores (>95%).

A few ADR prediction studies (3/36, 8%) introduced innovative ideas on feature preprocessing. Jeong et al [33] developed an ML prediction model in which the features are calculated from algorithms such as the “comparison of extreme laboratory test” results, “comparison of extreme abnormality ratio”, and “prescription pattern around clinical events” to help determine whether a drug-laboratory event pair is associated. A different approach was proposed by Wang et al [37] who addressed problems with low-quality observational data (eg, missing data) by creating patient embeddings and treating patients “as bags with the various number of feature-value pairs, called instances.” This method led to the development of the final AI model (AMI-Net3), which achieved exceptional performance. Chen et al [54] also proposed an embedding methodology, called “physiological signal embeddings.” This study proved that training deep embedding models on physiological signals could lead to better forecasts of adverse outcomes. In addition, this methodology enables data transferability through the physiological signal embeddings models.

Only 1 (3%) of the 36 studies developed an application for the prediction of ADRs. Mosa et al [41] leveraged the interoperability of a decision tree ML model and, based on their results, designed a rule-based mobile app to assess the risk of specific ADRs and indications.

A completely different approach to ADR prediction was introduced by Liu et al [26]. In this study, the authors applied an ML method to develop a prediction model for osteoarthritis ADRs in analgesic drugs. Afterward, the authors used explainability techniques to identify patients who might be prescribed analgesic drugs without the risk of osteoarthritis ADRs. The diversity of this study is addressed in a different scope: instead of predicting ADRs based on a patient’s medical history, the model focuses on identifying the characteristics that make the patient suitable for a medication, specifically by considering the presence or absence of an ADR.

Recently, the causal ML paradigm was introduced into pharmacovigilance through the studies of Wang et al [48] and Zhang et al [52], who applied causal inference with average treatment effects and causal discovery with directed acyclic graphs, respectively. Wang et al [48] used causal ML models to make a representation of a randomized clinical trial with EHR data. Their results successfully identified both well-known and new medications that could cause the suspected ADR in their case study. Furthermore, Zhang et al [52] created a causal graph for a drug-event combination and compared the results from 2 causal discovery algorithms. Their results showcased the causal discovery algorithms’ abilities to explore the mechanisms of the suspect drug that could lead to a potential ADR, uncovering previously unknown causal links.

Only 1 (3%) study focused on the use of symbolic AI [43] compared to those investigating the use of ML (35/36, 97%) and 1 study combine symbolic and nonsymbolic AI. Notably, Pichardo et al [43] stand out for integrating ontologies and ML, namely combining symbolic and nonsymbolic AI. The objective of this study is to examine the performance of a clinically informed framework for the prediction of short-term ADRs.

Furthermore, very few studies (5/36, 14%) focused on the clinical evaluation of the proposed ML approach. Segal et al [20] presented a clinical decision support system designed to provide medication error alerts to prevent ADRs, demonstrating significant results—40% of the prescriptions were altered based on these alerts. Herrin et al [42] compared the effectiveness of their proposed ML scheme to that of an established clinical practice, specifically the HAS-BLED approach, to evaluate a patient’s risk of gastrointestinal bleeding.

### Synthesized Findings

Although the included studies followed widely used approaches in AI and pharmacovigilance to predict potential ADRs, primarily using EHR data, such as predicting ADR outcomes using well-known ML model architectures, the aforementioned studies follow fundamental methodologies. In terms of data, it describes several interesting processes such as the creation of patient embeddings and the application of the “comparison of extreme laboratory test” results, “comparison of extreme abnormality ratio”, and “prescription pattern around clinical event” algorithms for calculating input data for the ML model. Furthermore, it is described a variety of innovative AI algorithms such as SCCS models, causal ML, and symbolic AI. Finally, out-of-the-box pharmacovigilance approaches were followed, such as identifying patients suitable for specific treatments based on their ADR profiles.

## Discussion

The findings of this study enabled us to identify innovative ideas, spot existing limitations, and propose potential directions for future work in this field.

### Principal Findings

In summary, the number of studies on AI methodologies applied to RWD for pharmacovigilance purposes has significantly increased in the last 5 years—most of the included studies (28/36, 78%) were published after 2019, with the United States contributing the most publications in this field (19/36, 53%).

Comparing this review study with 3 recent reviews from 2022 to 2023 in the same field, we conclude that only the study by Kaas-Hansen et al [55] could be considered a study with the exact focus with our review. Their review included only 7 scientific papers because they restricted their selection to studies published between 2015 and 2021 and involving >1000 patient records. Their findings are similar to ours in terms of dominant AI solutions (classification) and the type of RWD used (EHR). Finally, it is essential to note that their review, like ours, highlights the limited adoption of widely used common data models, such as the OMOP-CDM.

In terms of risk of bias, selection biases were due to the fact that most of the studies (15/36, 42%) did not include patients from >1 data source (regional hospital [46] or insurance claims database [24]) in their models. Regarding confounding, while high-dimensional RWD offer a significant amount of information, they also contain substantial noise and null values. Sequentially, features that could be potential confounders are eliminated from the final dataset.

### Pharmacovigilance

The reviewed papers focused on using ML to detect ADRs to confirm whether previously known ADRs could have been identified using RWD. Another major theme identified was the prediction of ≥1 ADRs based on the classification of patients with different characteristics, ultimately aiming to support personalized ADR prevention. However, there was a lack of studies investigating new, potential pharmacovigilance signals. Regarding the investigated ADRs, there was a slightly higher interest in chemotherapy drugs for different types of cancers due to the high incidence of serious reactions associated with this treatment.

Finally, it is important to highlight that only 4 (11%) of the 36 studies in this review were tested in real-world clinical environments, which leads us to conclude that AI models may lack generalizability or that health care professionals may lack trust in AI models. By contrast, the trustworthy AI evaluation based on the FUTURE-AI guidelines proved that only a few studies (4/36, 11%) failed to satisfy half of the criteria, indicating relatively high research quality.

### RWD Preprocessing

In terms of RWD, EHRs were the most commonly used data source. EHRs are multidimensional, offering data that could be crucial for detecting postmarketing ADRs. At least in principle, EHRs could serve as an invaluable data source for investigating potential drug synergies or interactions across diverse populations. Furthermore, the variety of information in patient records could be an advantage in the creation of multimodal datasets, for example, by integrating biological, signaling pathway, and drug information databases.

However, the use of EHRs comes with significant burdens because they contain sensitive personal data, leading to limited access. Medical Information Mart for Intensive Care is the only openly available EHR dataset for researchers, but it is not commonly used in pharmacovigilance (only 2/36, 6% studies used it).

Moreover, it is important to mention that RWD preprocessing is challenging due to its complexity and real-world nature (biases, errors, gaps, noise, etc). Consequently, less than half of the articles (17/36, 47%) described in detail the data preprocessing step in their pipelines.

Another noteworthy outcome is that widely adopted data models such as the OMOP-CDM, Informatics for Integrating Biology & the Bedside, and Sentinel appeared sporadically in the studies. This could be attributed to the fact that the use of EHR data and AI models is relatively new. However, it should be noted that initiatives in this direction are emerging (eg, the Assessment of Pre-trained Observational Large Longitudinal models in Observational Health Data Sciences and Informatics initiative [56]).

Finally, it should be noted that RWD have a very substantial longitudinal dimension and questionable quality (due to gaps, errors, etc). As such, leveraging RWD for pharmacovigilance purposes requires the development of new approaches that focus on using time-related sequential information. While several attempts have been made to exploit this temporal aspect of RWD [19,33-35], validating AI and ML algorithms focusing on time-series rationale for pharmacovigilance signal detection remains a critical issue.

### AI Models

The detection of potential pharmacovigilance signals is a challenging procedure. As a result, the development of ML models to support the detection of ADR signals could have a significant impact. We can outline 2 major approaches for ADR signal detection. The first focuses on creating an AI tool that could discover unknown relationships between drugs and conditions, highlighting potential causal associations. The second approach emphasizes AI pipelines tailored to specific ADRs, where the input data for the final ML model are preprocessed based on prior medical knowledge of the drug-event combination.

The AI models identified in this review are generally complex, with ensemble methods such as RF being the most commonly used. A significant number of studies also applied ANNs. As RWD contain a substantial amount of diverse information, the relationships between different features may not be linear. Hence, the use of black box models (eg, ensemble methods and ANNs) is essential for discovering more complicated associations in a dataset beyond linear relationships.

On the basis of the review papers, there is a noticeable lack of use of XAI models (ie, local interpretable model-agnostic explanations and Shapley additive explanations models). Health care professionals highlight the necessity to understand the motifs between AI models’ tasks to accept the decisions made by the algorithms. This could not only lead to the biological translation of the results based on existing knowledge but also reveal new information about a disease, a medication, and so on. In terms of pharmacovigilance, XAI models applied to RWD could bring evidence about unknown confounders in an ADR and provide more informative results for pharmacovigilance experts regarding the causes of a potential pharmacovigilance signal. Although XAI methods’ results are tested extensively in the health care domain, we found that only 6 recent studies (4/36, 11% are included in this review) had applied them in the pharmacovigilance domain [21,23,27,28,54,57] (4 in 2021, 2 in 2022). Another novel approach discussed extensively in the explainability field is the newly introduced causal ML or causal deep learning algorithm, which combines AI and causal inference to uncover underlying cause-and-effect relationships between variables. The complexity of RWD presents a challenge that causal ML could potentially solve more efficiently by providing meaningful explanations of the causal relationships between variables [58]. These innovative AI models could serve as a good hypothesis for future work because they seek and present the relationships between different variables in RWD sources with a more informative structure than traditional AI models. They have already been applied efficiently in pharmacological treatment patterns [59]. In this review, only 3 (8%) of the 36 studies applied causal deep learning to EHR data [25,48,52].

Finally, a major problem identified based on the SR findings is the lack of code availability. This issue hinders the reproducibility of the models, preventing further testing on different datasets and raising questions about the developed AI models’ robustness and generalizability.

### Strengths and Limitations

The strengths of this review include the use of a considerable number of studies (n=36), providing a thorough knowledge of the specific scientific field. Besides, we compared our findings with those of the most recent review in the same field and analyzed the differences.

Nevertheless, this systematic review has several limitations. First, we only included articles from the MEDLINE database. As such, we may have excluded other existing AI approaches to structural RWD in the field of pharmacovigilance that could be available in AI databases. Second, because of the variation in the articles’ methodologies, we were unable to conduct a meta-analysis of the quantitative results. Finally, it is important to mention the limited number of symbolic AI studies in this review (4/36, 11%) [17,45,60,61]. The construction of knowledge graphs (KGs) usually requires the use of text mining procedures such as NLP and focused on real text such as clinical notes. As we excluded NLP studies from our query, we assume that this contributed to the small number of symbolic AI articles (n=36) included in our review.

### Current Gaps and Potential Future Work Paths

Detecting new pharmacovigilance signals using ML approaches requires evidence of a causal association between the suspect drug and the reaction. XAI models can assist pharmacovigilance professionals in this process. To this end, further investigation into causal ML and causal deep learning approaches could be a highly impactful line of research for identifying pharmacovigilance signals from RWD.

Another gap identified in this SR that could indicate future work paths could be the use of multitask learning approaches. Multitask learning is an ML methodology that takes as input 1 dataset to execute multiple prediction tasks. RWD, such as EHR data, are rich data sources that could support >1 task (eg, pharmacovigilance and pharmacoepidemiology); for instance, a multitask learning model could predict an adverse drug event, the severity of an adverse drug event, and the likelihood of the same adverse drug event occurring with other drugs in a patient.

Furthermore, combining ML approaches with symbolic AI is a line of work that offers further potential for exploration. Combining ML with ontologies and automatic reasoning upon KGs could enable new AI approaches (eg, neurosymbolic AI) and provide new insights based on well-established expert knowledge formed as a KG. Moreover, using ontologies and KGs could support integration with other kinds of data sources (eg, data sources containing low-level biochemical or pharmacokinetics and pharmacodynamics information and signaling pathway information).

Finally, exploiting the currently formed federated data networks could also be an interesting area for future research; for example, the European Health Data & Evidence Network is currently setting up a network of >180 data partners across Europe, using the OMOP-CDM as the main data model [62]. The adoption of the OMOP-CDM and the potential exploitation of such data networks would significantly enhance the prospects of potential AI models used for pharmacovigilance.

### Conclusions

In this paper, we reviewed scientific papers focusing on AI approaches to structured RWD for pharmacovigilance purposes. It should be noted, as a key finding, that most models are designed not for pharmacovigilance signal detection but for personalized ADR prediction. Furthermore, XAI methods and causal ML and causal deep learning are not investigated in depth. Moreover, there are no identified gold standard methodologies for data preprocessing of structured RWD for pharmacovigilance. Finally, an evaluation of the already developed AI models in external data is difficult because of code unavailability and a lack of data access.

Therefore, there is an essential need for more informative XAI models that can be validated on external datasets and for a more detailed description of RWD preprocessing pipelines and methods to examine potential pharmacovigilance signals in clinical practice. Implementing AI approaches in RWD analysis could tackle the problems of pharmacovigilance signaling underreporting and support the vision of personalized ADR management.

## Appendix

Multimedia Appendix 1 Supplementary tables.

Multimedia Appendix 2 PRISMA-ScR checklist.

## Abbreviations

ADR: adverse drug reaction
AI: artificial intelligence
ANN: artificial neural network
ConvSCCS: convolutional self-controlled case series
DARWIN: Data Analysis and Real World Interrogation Network
EHR: electronic health record
FUTURE-AI: Fairness, Universality, Traceability, Usability, Robustness, and Explainability–Artificial Intelligence
ICSR: individual case safety report
KG: knowledge graph
ML: machine learning
NLP: natural language processing
NSCCS: neural self-controlled case series
OMOP-CDM: Observational Medical Outcomes Partnership Common Data Model
PRISMA: Preferred Reporting Items for Systematic Reviews and Meta-Analyses
PRISMA-ScR: Preferred Reporting Items for Systematic Reviews and Meta-Analyses extension for Scoping Reviews
RF: random forest
RWD: real-world data
SCCS: self-controlled case series
XAI: explainable artificial intelligence

## Data Availability

All data generated or analyzed during this study are included in this published paper and its supplementary information files.
